# Multidrug-Resistant *Shigella sonnei* Bacteremia among Persons Experiencing Homelessness, Vancouver, British Columbia, Canada

**DOI:** 10.3201/eid2908.230323

**Published:** 2023-08

**Authors:** A. Stefanovic, N. Matic, G. Ritchie, C.F. Lowe, V. Leung, M. Hull, M. Alam, M. Dawar, S. Champagne, M.G. Romney

**Affiliations:** St. Paul’s Hospital, Vancouver, British Columbia, Canada (A. Stefanovic, N. Matic, G. Ritchie, C.F. Lowe, V. Leung, S. Champagne, M.G. Romney);; University of British Columbia, Vancouver (A. Stefanovic, N. Matic, G. Ritchie, C.F. Lowe, V. Leung, M. Hull, M. Alam, S. Champagne, M.G. Romney);; British Columbia Centre for Excellence in HIV/AIDS, Vancouver (M. Hull);; Vancouver Coastal Health Authority, Vancouver (M. Dawar);; University of British Columbia School of Population and Public Health, Vancouver (M. Dawar)

**Keywords:** Shigella sonnei, shigellosis, bacteria, antimicrobial resistance, enteric infections, bacteremia, persons experiencing homelessness, sexually transmitted infections, Canada

## Abstract

Increased invasive bloodstream infections caused by multidrug resistant *Shigella sonnei* were noted in Vancouver, British Columbia, Canada, during 2021–2023. Whole-genome sequencing revealed clonal transmission of genotype 3.6.1.1.2 (CipR.MSM5) among persons experiencing homelessness. Improvements in identifying *Shigella* species, expanding treatment options for multidrug resistant infections, and developing public health partnerships are needed.

Shigellosis manifestations range from mild gastrointestinal infection to severe illness with dysentery and sepsis (*1*). In high-income countries, *Shigella sonnei* is the most common species, causing infections typically among men who have sex with men (MSM) and travelers (*1*,*2*). Transmission occurs through sexual contact in MSM or the fecal–oral route from contaminated water, food, or fomites (*3*). Although clinical manifestations range broadly, *S. sonnei* rarely causes invasive bloodstream infections. Only a few published case reports describe bacteremia (*4*–*6*), mostly among malnourished children, MSM, or adults with HIV, diabetes, cirrhosis, or immunosuppression (*4*,*6*,*7*). We describe the epidemiology, genotyping, and resistance determinants of clonal multidrug-resistant (MDR) *S. sonnei* bacteremia in Vancouver, British Columbia, Canada, and discuss challenges in diagnosing *Shigella* bacteremia in the microbiology laboratory. The University of British Columbia/Providence Health Care Research Ethics Board approved our study (H22–02183). 

## The Study

The microbiology laboratory at St. Paul’s Hospital (Vancouver, BC, Canada) serves acute-care hospitals and the surrounding community in downtown Vancouver. We searched the laboratory database for *S. sonnei* found in feces and blood samples during January 2010–January 2023, separated into 2010–2020 (historical) and 2021–2023 (recent) periods. We reviewed medical records of patients with bacteremia and recorded demographics, symptoms, housing, sexual orientation, travel, substance use, coexisting conditions, hospitalization, antimicrobial susceptibility testing (AST), treatment, and mortality. 

We processed positive blood cultures detected by BacT/AlertT system (bioMérieux, https://www.biomerieux.com) using VitekMS+ (bioMérieux) or FilmArray BCID (BioFire Diagnostics; https://www.biofiredx.com) with established microbiology protocols and identified pathogens. We identified *Shigella* in feces using benchtop biochemical methods, Vitek2 ID (bioMérieux) and Polyvalent Agglutination Sera (Remel, http://www.remel.com). If we suspected *Shigella* in blood samples, we used Vitek2 ID and polyvalent serology. No changes in laboratory testing protocols occurred during 2010–2023. We performed AST for ampicillin, trimethoprim/sulfamethoxazole, ciprofloxacin, ceftriaxone, and azithromycin according to Clinical and Laboratory Standards Institute M100 standards (https://clsi.org/standards/products/microbiology/documents/m100). We performed whole-genome sequencing (WGS) on isolates incubated in Mueller-Hinton broth, extracted on MagNA Pure 24 (Roche; https://diagnostics.roche.com), and processed on GridION R10.4 flowcells (https://nanoporetech.com). We basecalled runs with Guppy version 6.3.9 (https://github.com/nanoporetech/rerio) and uploaded to BugSeq (https://bugseq.com) for automated analysis. 

We identified 11 cases of *S. sonnei* bacteremia that occurred within the historical (n = 2) or recent (n = 9) periods during 2010–2023. We also observed a recent increase in fecal isolates with *S. sonnei* (Figure). Differences in the proportion of bacteremic among all shigellosis cases occurring during the recent compared with the historical period were not statistically significant (7.7% vs. 2.9%; p = 0.21 by Fisher exact test). Among recent cases, 89% (8/9) of patients were male (median 45 years of age, interquartile range 35–54 years) (Table). All but 1 were underhoused or experiencing homelessness and had polysubstance use disorder. Most (89%) inhabited Vancouver’s downtown eastside, the neighborhood with the highest density of Vancouver’s urban poor population. Unlike the historical case-patients, none were MSM or had travel histories. Most (89%) were not severely immunocompromised. Case-patient A had multiple myeloma but stable housing. Five (55%) patients presented with sepsis and 6 (67%) were hospitalized; the remaining patients declined recommended hospital admission. 

**Figure:**
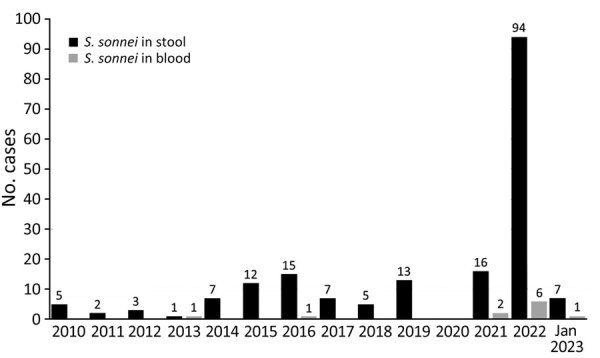
Epidemic curve of *Shigella sonnei* cases from feces and blood, Vancouver, British Columbia, Canada, 2010–January 2023.

**Table:** Characteristics and outcomes of cases of *Shigella sonnei* bacteremia during recent and historical time periods, Vancouver, British Columbia, Canada*

Characteristic	Cases of *Shigella sonnei* bacteremia	
	Recent, 2021–Jan 2023, n = 9	Historical, 2010–2020, n = 2
Age, y	Median 45, range 27–69	Median 62, range 56–68
Sex	8 (89%) male	2 (100%) male
Housing		
Private residence	1 (11)	2 (100)
Single room occupancy hotel/shelter	5 (56)	0
No fixed address	2 (22)	0
Rehabilitation center	1 (11)	0
Men who have sex with men	0	2 (100)
Travel history	0	1 (50)
Substance use	8 (89)	0
Coexisting conditions		
HIV	1 (11)	1 (50)
Immunocompromising conditions†	1 (11)	1 (50)
Liver disease	2 (22)	0
Pulmonary disease	1 (11)	0
Cardiac disease	1 (11)	0
Chronic renal disease	0	1 (50)
Recurrence	1 (11)	0
Hospitalization		
Admitted to hospital ward	6 (67)	2 (100)
Declined hospital admission	3 (33)	0
Antimicrobial test results		
Ampicillin resistant	9 (100)	2 (100)
Trimethoprim/sulfamethoxazole resistant	9 (100)	2 (100)
Ciprofloxacin resistant	9 (100)	0
Azithromycin resistant	8 (89)	Not tested
Ceftriaxone resistant	1 (11)	0
Treatment		
7–14 d of effective oral antimicrobial agent	0	1 (50)
7–14 d of effective IV antimicrobial agent	6 (67)	1 (50)
Incomplete	3 (33)	0
30-d all-cause mortality	0	0

*Values are no. (%) except as indicated. IV, intravenous. †Organ transplantation, malignancy receiving chemotherapy, asplenia, uncontrolled HIV (CD4 count <200 cells/μL), and immunosuppressive medications.

Among the 2021–2023 cases, AST profiles were identical except in case A. The isolates were resistant to ampicillin, trimethoprim/sulfamethoxazole, ciprofloxacin, and azithromycin and susceptible to ceftriaxone. An isolate from case-patient A displayed ceftriaxone resistance and azithromycin susceptibility. *Shigella* was initially misidentified as *E. coli* in all 9 cases (8 by VitekMS+, 1 by FilmArray BCID). All isolates were indole negative, non–lactose fermenting (NLF) colonies, subsequently identified correctly as *S. sonnei* by Vitek2 ID and confirmed by polyvalent serology. 

We performed WGS on all 2021–2023 *S. sonnei* isolates from blood samples. Using a new genotypic framework (*8*), we identified 8 of the 2021–2023 isolates as 3.6.1.1.2 (CipR.MSM5); the isolate from case-patient A genotyped as 3.6.3 (Central Asia III). The strain from the 2016 case genotyped as 3.7.18 (Global III); the 2013 strain did not undergo WGS. We identified mutations *gyrA* S83L, *gyrA* D87G, and *parC* S80I encoding ciprofloxacin resistance and plasmid AA336-borne *mphA* and *ermB* encoding azithromycin resistance in all isolates from persons experiencing homelessness (PEH). The 3.6.3 strain carried *qnrS1, gyrA* D87Y, *gyrA* S83L and *parC* S80I, which confers ciprofloxacin resistance, and *bla*_CTX-M-15_, which confers ceftriaxone resistance. 

## Conclusions

The recent increase in *S. sonnei* bacteremia might reflect the overall increase in shigellosis, including *S. sonnei* isolated from feces. The increased proportion of bacteremia cases in the past 2 years compared with the 11-year historical period was not statistically significant. However, the 7.7% prevalence of bacteremia is still very high compared with a range of rates, 0.4%–7.3%, reported in the literature (*9*). Historically, shigellosis has occurred in British Columbia predominantly as a sexually transmitted enteric infection among MSM (*10*). In our study, invasive shigellosis among PEH was probably transmitted fecal-orally through contaminated environment and hands. An outbreak among PEH in Oregon was similarly believed to have resulted from inadequate access to hygiene and sanitation (*11*). WHO warns of outbreak risk from *S. sonnei* being introduced into areas with suboptimal water, sanitation, and hygiene standards (*12*). Because patient immunosuppression fails to explain increased bacteremia, other factors, such as drug use, malnutrition, and high inoculum dose, should be considered.

A study in Seattle, Washington, USA described a contemporary increase in MDR *S. sonnei* cases among PEH (*13*). Although the report did not comment on bacteremia, it highlighted the circulation of extensively-drug resistant *S. sonnei* carrying the extended-spectrum β-lactamase CTX-M-27. Although extensively-drug resistant *S. sonnei* was rare in our review, increased hospitalization and the need for parenteral therapy because of MDR *S. sonnei* bacteremia still substantially affected the healthcare system. One third of our patients did not complete treatment, potentially leading to ongoing transmission and illness. In addition to finding and treating cases, essential community control measures include working with housing providers to promote handwashing and sanitation practices, providing advice on recognizing and controlling infectious diarrhea, and developing pathways to reengage those who refuse hospitalization to complete parenteral antimicrobial treatment as outpatients.

Isolates from 8/9 PEH were genotype 3.6.1.1.2 (CipR.MSM5/BAPS3), epidemiologically distinct from the single isolate typed as 3.6.3 (Central Asia III). The isolate from the 2016 case had a different genotype, 3.7.18, matching that from an outbreak reported in California, USA (*14*). Despite its geographic proximity to Vancouver, the recent *S. sonnei* outbreak among PEH in Seattle involved yet another different genotype, 3.7.29.1.4.1 (global III VN2.KH1.Aus) (*14*). The 3.6.1.1.2 strain predominant in our study has previously been described in Australia, England, and the United States (*8*). 

Our study included analysis of data only from patients seeking treatment at a hospital where blood cultures were collected, possibly representing the sickest cohort of patients, leading to underestimation of actual infections. Not all cases with *S. sonnei* isolated from feces have yet been analyzed to determine wider epidemiologic and antimicrobial resistance trends. Further studies are needed to elucidate whether the high rate of bacteremia reflects increased virulence of this strain, higher inoculum size, or host determinants. 

Because current laboratory methods can misidentify *Shigella* as *E. coli* in bloodstream infections, laboratories must scrutinize diagnoses of *E. coli* bacteremia in high-risk patients with infectious diarrhea and sepsis. Clinicians and public health officials should be made aware of MDR *S. sonnei* bacteremia as a cause of increased illness among PEH and the need for parenteral therapy in the event of resistance to first- and second-line antimicrobial agents. 
